# Concurrent Increases in Leaf Temperature With Light Accelerate Photosynthetic Induction in Tropical Tree Seedlings

**DOI:** 10.3389/fpls.2020.01216

**Published:** 2020-08-07

**Authors:** Hui-Xing Kang, Xin-Guang Zhu, Wataru Yamori, Yan-Hong Tang

**Affiliations:** ^1^ Institute of Ecology, College of Urban and Environmental Sciences and Key Laboratory for Earth Surface Processes of Ministry of Education, Peking University, Beijing, China; ^2^ Center of Excellence for Molecular Plant Sciences and State Key Laboratory of Plant Molecular Genetics, Chinese Academy of Sciences, Shanghai, China; ^3^ Institute for Sustainable Agro-Ecosystem Services, Graduate School of Agricultural and Life Sciences, The University of Tokyo, Tokyo, Japan

**Keywords:** dynamic photosynthesis, photosynthetic induction, Rubisco, shade tolerance, sunflecks, temperature

## Abstract

Leaf temperature changes with incident light intensity, but it is unclear how the concurrent changes influence leaf photosynthesis. We examined the time courses of CO_2_ gas exchanges and chlorophyll fluorescence of seedling leaves in four tropical tree species in response to lightflecks under three different temperature conditions. The three conditions were two constant temperatures at 30°C (*T*_30_) and 40°C (*T*_40_), and a simulated gradually changing temperature from 30 to 40°C (*T*_dyn_). The time required to reach 50% of the full photosynthetic induction under *T*_40_ was similar to, or even larger than, that under *T*_30_. However, the induction of assimilation rate (*A*) and electron transport rate of photosystem II (ETR II) and Rubisco activation process were generally accelerated under *T*_dyn_ compared to those at either *T*_30_ or *T*_40_. The acceleration in photosynthetic induction under *T*_dyn_ was significantly greater in the shade-tolerant species than in the shade-intolerant species. A modified photosynthetic limitation analysis indicated that the acceleration was likely to be mainly due to ETR II at the early stage of photosynthetic induction. The study suggests that concurrent increases in leaf temperature with light may increase leaf carbon gain under highly fluctuating light in tropical tree seedlings, particularly in shade-tolerant species.

## Introduction

Most of our understanding on plant photosynthesis so far is almost completely based on the measurements made under so-called steady-state or temporally constant environments. However, photosynthesis in nature rarely or even never occurs under constant environments, but under fluctuating light, and changing temperature and other environmental variables. Field observations showed considerable variation in photosynthetically active radiation (PAR) at different temporal scales from seconds to days under tropical forest canopies (Pearcy, 1983; Tang et al., 1999). Efficient utilization of temporally variable light has been considered to be critical for leaf carbon gain (Pearcy, 1990; Kaiser et al., 2015; Tomimatsu and Tang, 2016; Yamori, 2016).

Temporal changes in PAR under forest canopies are often accompanied with changes in leaf temperature (*T*_leaf_; Singsaas and Sharkey, 1998; Wise et al., 2004). Changes in *T*_leaf_ can be closely associated with changes of PAR. For example, leaf temperature increased from 32 to 39°C within several min due to sunflecks (Leakey et al., 2003). Despite of a limited number of observations indicating a close relationship between changes in *T*_leaf_ and changes in light intensity, there is no detailed quantitative description, within our knowledge, for *T*_leaf_ changes in response to a step change in light intensity. Nonetheless, such associated changes in temperature with light are expected to influence photosynthesis in nature because leaf photosynthesis is a highly temperature-dependent process (Berry and Björkman, 1980). Recent studies further suggest that photosynthetic induction in response to an increase in PAR varied at different constant temperatures (Leakey et al., 2003; Kaiser et al., 2017; Wachendorf and Küppers, 2017). Moreover, thermal responses of photosynthesis are highly species specific (Slot et al., 2016; Slot and Winter, 2017a; Slot and Winter, 2017b; Fauset et al., 2018). However, very little knowledge has been accumulated regarding concurrent changes in leaf temperature with light on dynamic photosynthesis, despite the fact that the changes may be potentially important for leaf carbon gain under fluctuating light and temperature conditions in nature.

In this study, we characterized induction kinetics in four lowland tropical tree species under two constant temperatures and a simulated dynamic temperature condition, aiming to address (1) how the concurrent changes in leaf temperature with light affect the photosynthetic induction process, (2) if and how major physiological and biochemical processes contribute to the effect(s), and (3) whether there are any differences in the effect(s) between shade-tolerant and shade-intolerant tree species in tropical rain forests.

## Materials and Methods

### Study Site and Plant Species

The study was conducted in a lowland tropical rain forest in Pasoh Forest Reserve (2°59′N, 102°08′E), Malaysia. This is a primary Dipterocarp forest with an averaged leaf area index estimated as 6.52 in the core area of the reserve (Tani et al., 2003b). The annual rainfall of the normal years, i.e., no El Niño years, observed by the meteorological station within the reserve averaged 1809 mm during the period from 1983 to 1990. Most rainfall was observed during the rainy season from March to May and from October to December. Mean annual temperature at 52 m above the forest floor was 25.6°C, ranging from 22.6 to 29.9°C (Tani et al., 2003a).

The study species were two shade-intolerant species, *Croton argyratus* Blume and *Shorea leprosula* Miq., and two shade-tolerant species, *Neobalanocarpus heimii* (King) Ashton and *Lepisanthes senegalensis* (Poir.) Leenh, which are all native to lowland forests (Thomas et al., 2003). Five to six seedlings from different light regimes were selected for each species. Light regime was characterized as averaged daily light integral (DLI) of 60 days prior to the experiment (unit mol m^-2^ d^-1^), which was estimated from hemispherical photographs using SOLARCALC 7.0 (Mailly et al., 2013). All field measurements were conducted between August and October 2018.

### Leaf Gas Exchange and Chlorophyll Fluorescence

Photosynthetic induction responses were measured using a LI-6800 (LI-COR, Lincoln NE, USA) fitted with a LI-6800-01 fluorometer (90% red and 10% blue) on a fully expanded and healthy leaf in each selected seedling. Leaves were first acclimated to the irradiance at 50 μmol m^-2^ s^-1^ for at least 20 min until steady-state assimilation rate (*A*) and stomatal conductance for H_2_O (*g*_sw_) were visibly reached, after which light was raised to 1000 μmol m^-2^ s^-1^ for 32 min. *A*, *g*_sw_, and intercellular CO_2_ concentration (*C*_i_) were logged every second. To avoid any artefacts from correctional changes in temperature or relative humidity, temperature of the heat exchanger (*T*_exchg_) was controlled. Photosynthetic induction was measured under three different temperature conditions, i.e., two constant temperature conditions with 30°C (*T*_30_) and 40°C (*T*_40_), and a simulated dynamic temperature condition (*T*_dyn_). For the two constant temperatures, *T*_leaf_ reached a constant value around 30.7°C under *T*_30_ and 36.6°C under *T*_40_ prior to the increase in light. Under the *T*_dyn_ condition, *T*_exchg_ was kept at 30°C before the increase in light and then set to an expected value of 40°C at the same time when light increased. The warming speed of leaf temperature was similar to our observation within the same forest (**Figure S1**). Prior to the induction, leaf-to-air vapor-pressure deficit (VPD) was kept steady around 1 kPa under *T*_dyn_ and *T*_30_ and 2.3 kPa under *T*_40_ to mimic the natural levels at each temperature, according to our records of within-canopy microenvironments (see **Figure S2**). Reference CO_2_ concentration was maintained at 400 μmol mol^-1^. Photosynthetic CO_2_ response curves were generated with a LI-6400XT equipped with a LI-6400-02B LED light source on the same leaves at a block temperature of 30 and 40°C. Leaves were first fully induced under 400 μmol mol^-1^ and 1000 μmol m^-2^ s^-1^. Then, the reference CO_2_ concentration was reduced to 50 μmol mol^-1^ in a stepwise manner, after which it returned to the starting level. When steady-state *A* was again reached, the CO_2_ concentration was increased to 1500 μmol mol^-1^ in several steps. Flow rate was maintained at 350 μmol s^-1^, and relative humidity was controlled at 70%, which yield a VPD similar to that reached at the end of induction.

All measurements were repeated with the same environmental settings as the measurement of photosynthetic induction course to produce the time courses of chlorophyll fluorescence signals using the same LI-6800. Hence, we obtained two sets of induction curves, one with gas exchange only and the other with both gas exchange and chlorophyll fluorescence. Leaf samples were placed in dark for at least 2 h. Then, light was increased to 50 μmol m^-2^ s^-1^ until gas exchange parameters reached steady state, which typically took 20 min, followed by 30 min of induction. However, due to weather and insufficient time, some chlorophyll fluorescence measurements under *T*_dyn_ started from a light intensity of 50 μmol m^-2^ s^-1^ directly without dark adaptation. For these measurements, less time (~10 min) was required to reach steady state under low light. Gas exchange parameters were recorded every 5 s, and chlorophyll fluorescence was recorded every minute. Recorded chlorophyll fluorescence signals include *F*_o_ and *F*_M_, if leaves were dark adapted, *F*_s_, *F*_M_’, and *F*_o_’ by turning off the actinic light and then applying far-red light. We used the multi-phase flash (MPF) protocol of the fluorometer for measuring *F*_M_ and *F*_M_’. MPF settings were as factory default, including 8000 μmol m^-2^ s^-1^ for flash beam intensity, 40% ramp reduction during the 2^nd^ phase of the MPF, and 0.3 s duration of each flash phase. The quantum yields of photosystem II [*Y*(II)] were calculated after Yamori et al. (2012). The electron transport rates of photosystem II (ETR II) were calculated using the following equation: ETR II = 0.5 × *α* × *I* × *Y*(II), where 0.5 is the fraction of absorbed light allocated to photosystems II, *α* is leaf light absorptance (see below), and *I* is light intensity. The quantum yields of photochemical quenching based on the puddle (qP) and the lake model (qL) and non-photochemical quenching (NPQ) were calculated as described by Kalaji et al. (2017). Data obtained without dark adaptation were excluded from NPQ calculation.

### Light Absorptance

Leaf light absorptance was calculated from measured reflectance and transmittance. For each species, four to six branches from seedlings other than those for photosynthesis measurements were sampled around 18:00 h, with the cut end submerged in water immediately. Samples were kept in dark and then measured within 6 h using a Maya-2000-Pro spectrometer (Ocean Optics, Dunedin, FL, USA). Four to six healthy, fully expanded leaves in each sampled branch and three to four discs per leaf were measured. Light absorptance was calculated with respect to the irradiance spectrum of the LI-6800-01 fluorometer, which was also measured with the same spectrometer. This yield leaf light absorptance of 0.88, 0.87, 0.90, and 0.92 for *N. heimii*, *L. senegalensis*, *C. argyratus*, and *S. leprosula*, respectively.

### Data Analysis

For those measurements made under *T*_dyn_, the time course of H_2_O concentration in the sample cell (H_2_O_s_) exhibited an unusually steep peak within the first minute, since the LI-6800 started to elevate *T_e_*_xchg_. As a result, stomatal conductance doubled and *C*_i_ increased during the first minute since LED light and *T*_exchg_ concurrently changed. After excluding the possibility of a contaminated leaf chamber by repeating the same measurement settings with a brand new LI-6800 later, we suspected that such errors were induced by the heat exchanger itself. We matched the LI-6800 only immediately before each measurement, and the differences in match adjustment factor between two consecutive measurements were small compared to the differences in water concentrations result from foliar transpiration. Thus, we proposed an empirical method to sequentially correct H_2_O_s_, transpiration rate, *A*, *g*_sw_, and *C*_i_ (for detailed information, see **Supplementary File S1**).

To determine the maximum rate of increase in $A\;\left({\frac{dA}{dt}}_{\max}\right)$, the time courses of *A* during induction (gas exchange only) were fitted to the Boltzmann sigmoidal model proposed by Drake et al. (2013):

(1) $$A(t)=\frac{a_{1}-a_{2}}{1+e^{\left(t-t_{0}\right)}/\Delta t_{A}}+a_{2}$$

where *a*_1_ and *a*_2_ are the left and right horizontal asymptotes, respectively, *t*_0_ is the point of inflection, and Δ*t_A_* describes the steepness of the curve. The maximum rate of increase is the value of the derivative of Equation (1), where *t* = *t*_0_. The maximum rate of increase in $g_{sw}\left({\frac{dg}{dt}}_{\max}\right)$ was calculated in the same way.

To assess if sunfleck utilization was improved or inhibited under *T*_dyn_ and *T*_40_, induction carbon gain (ICG) at time *t* was calculated after Chazdon and Pearcy (1986a):

(2) $$\text{ICG}(t)=\int_{0}^{t}A(t)dt-t*A_{\text{ini}}$$

where *A*_ini_ is the steady-state *A* prior to the induction.

To identify the transition point between Rubisco and RuBP regeneration limitation (*C*_i,trans_) at high temperature, photosynthetic CO_2_ response curves were fitted after Bellasio et al. (2016), assuming a constant *R*_L_ *: R*_d_ ratio of 60% (Way et al., 2019). *R*_d_ was calculated by averaging the readings over the last minute in the dark period during chlorophyll fluorescence measurements. *C*_i,trans_ was determined as:

(3) $$C_{\text{i,trans}}=\frac{8\Gamma^{*}V_{\text{c,max}}-K_{m}J_{1000}}{J_{1000}-4V_{\text{c,max}}}$$

where *V*_c,max_ is the apparent maximum carboxylation rate of Rubisco, *J*_1000_ is the potential electron transport rate under 1000 μmol m^-2^ s^-1^, Г^*^ is the CO_2_ photocompensation point, and *K*_m_ is the effective Michaelis-Menten constant for Rubisco after Bernacchi et al. (2001).

To obtain the apparent time constant of Rubisco activation (τ_Rubisco_), transient *A*, recorded during chlorophyll fluorescence measurements, was corrected to steady-state *C*_i_ reached at the end of induction (*C*_i,f_) with respect to transient *T*_leaf_ after Urban et al. (2007) and then fitted to the exponential function proposed by Woodrow and Mott (1989):

(4) $$A^{*}(t)=A_{f}^{*}-(A_{f}^{*}-A_{i})*\exp(-t/\tau_{\text{Rubisco}})$$

where $A_{f}^{*}$ is the final corrected *A* and *A_i_* is the estimated initial *A* prior to the induction. For modeling convenience, we assumed that Rubisco is a one-phase process and used the data from whole induction curves for fitting. In the prior test, we found that fitting the whole curves yield higher *R* ^2^ and smaller confidence intervals than only fitting the data from minute 2 to 10 after the light increase in 25 among 30 cases. We also acknowledge that using the data from whole curve could underestimate *τ*_Rubisco_. Using transient *C*_i_ recorded during chlorophyll fluorescence measurements, we calculated the potential *A* supported by transient ETR II (*A*_j_) and that supported by transient carboxylation rate with respect to transient *T*_leaf_ (*A*_c_):

(5) $$A_{j}(t)=\text{ETR}(t)\frac{C_{i}(t)-\Gamma^{*}(T)}{4C_{i}(t)+8\Gamma^{*}(T)}-R_{L}(T)$$

(6) $$A_{c}(t)=V_{c}(t)\frac{C_{i}(t)-\Gamma^{*}(T)}{C_{i}(t)+K_{m}(T)}-R_{L}(T)$$

The temperature response of *R*_d_ was described for each leaf studied using an exponential model with *Q*_10_ (Vanderwel et al., 2015). The temperature dependency of Г^*^ for each leaf was described by the Arrhenius function using the CO_2_ response curves:

(7) $$\Gamma^{*}(T)=\Gamma^{*}(25)*\exp\left[\frac{E_{a}*10^{3}(T-298.15)}{298.15*R*T}\right]$$

where Г^*^(25) is Г^*^ at 25°C and *E_a_* is the activation energy term. *R* is the molar gas constant. For simplicity, we assume that *R*_L_, *K*_m_, and Г^*^, which respond to fluctuations in temperature instantaneously, and components of ETR II, i.e., fraction of absorbed light allocated to photosystems II and leaf light absorptance, remain constant during induction. Considerations of these assumptions are described in detail in Discussion. Transient carboxylation rate (*V*_c_) was estimated in analogy to Eqn. (1):

(8) $$V_{c}(t)=V_{\text{c,f}}-(V_{\text{c,f}}-V_{\text{c,ini}})*\exp(-t/\tau_{\text{Rubisco}})$$

*V*_c,f_ and *V*_c,ini_ were estimated from the so-called one-point method (De Kauwe et al., 2016) using data recorded before and at the end of induction, respectively. Assimilation rate decreased during induction in some measurements made under *T*_dyn_.

ETR II obtained under photorespiratory condition was likely to deviate from true linear electron transport rate, leading to incorrect *A*_j_. Considerations on how to model the midway decrease in *A* during induction and necessary calibration of ETR II are described in detail in **Supplementary File S2**. We compared *A*_c_(*t*) against *A*_j_(*t*) to determine whether photosynthetic rate was limited by Rubisco carboxylation or RuBP regeneration at time *t*.

### Statistical Analysis

To determine the effects of measurement temperature condition, data were compared by one-way ANOVA test. Data were log-transformed to meet the assumptions of normality and homogeneity of variances when necessary. Otherwise, a non-parametric Kruskal-Wallis test was used. All tests were conducted using SPSS Statistics Version 20.0 (IBM Corp., New York, USA). To examine whether the variances in the induction responses between *T*_30_ and *T*_dyn_ were related to species-specific shade tolerance (*S*) and DLI, we performed a two-way ANOVA analysis using *S* and DLI as the main factors and *S* × DLI as the interaction factor. The differences in induction responses were represented as the percentage change of a parameter. These tests were carried out in R version 3.5.0 (R Core Team, 2018).

## Results

### Photosynthetic Induction Response

**Figure 1:**
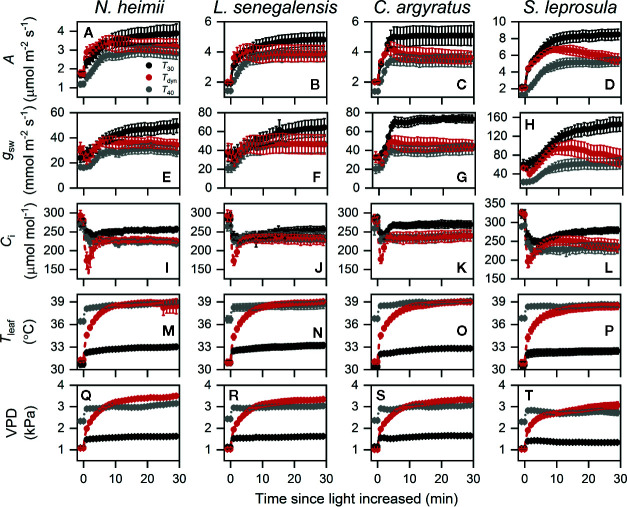
Time courses of *A* **(A–D)**, *g*_sw_ **(E–H)**, *C*_i_ **(I–L)**, *T*_leaf_ **(M–P)**, and VPD **(Q–T)** during photosynthetic induction in *N. heimii* **(A, E, I, M, Q)**, *L. senegalensis* **(B, F, J, N, R)**, *C. argyratus* **(C, G, K, O, S)**, and *S. leprosula* **(D, H, L, P, T)**. Shown are the data recorded during gas exchange only measurements under constant 30°C (*T*_30_) and 40°C (*T*_40_) and simulated dynamic temperature condition (*T*_dyn_). Values are the means ( ± SE) of five to six individual seedlings for each species. *A*, assimilation rate; *g*_sw_, stomatal conductance for H_2_O; *C*_i_, intercellular CO_2_ concentration; *T*_leaf_, leaf temperature; VPD, leaf-to-air vapor pressure deficit.

Time courses of photosynthetic induction under three different temperature conditions are shown in **Figure 1**. After full acclimation under *T*_40_, both initial photosynthetic rate (*A*_ini_) and final steady-state photosynthetic rate (*A*_f_) were significantly smaller than those under *T*_30_ (**Table 1**). The maximum rate of increase in $A\left({\frac{dA}{dt}}_{\max}\right)$ under *T*_40_ decreased by 31–64% compared to that under *T*_30_.

**Table 1:** Parameters of photosynthetic induction since the increase in irradiance from 50 to 1000 μmol m^-2^ s^-1^ in four tropical woody species under constant 30°C (*T*_30_), 40°C (*T*_40_), and simulated dynamic temperature condition (*T*_dyn_). *A*_ini_, *A*_f_, *g*_sw,ini_, *g*_sw,f_, *C*_i,ini_, and *C*_i,f_ were *A*, *g*_sw_, and *C*_i_ reached before and at the end of photosynthetic induction, respectively, calculated by averaging single values over the last minute of each period; IT_f50%_, the time required to reach 50% of the difference between *A*_ini_ and *A*_f_; ${\frac{dA}{dt}}_{\max}$ and ${\frac{dg}{dt}}_{\max}$ were the maximum increasing rate of *A* and *g*_sw_, respectively.

Species	Temperature	*A*_ini_	*A*_f_	*g*_sw,ini_	*g*_sw,f_	*C*_i,ini_	*C*_i,f_	IT_f50%_	${\frac{dA}{dt}}_{\max}$	${\frac{dg}{dt}}_{\max}$
Abbreviation	Condition	(μmol m^-2^ s^-1^)		(mmol m^-2^ s^-1^)		(μmol mol^-1^)		(s)	(μmol m^-2^ s^-2^)	(mmol m^-2^ s^-2^)
*C. argyratus*	*T*_30_	1.99 ± 0.03a	5.09 ± 0.60a	32.0 ± 1.9a	73.1 ± 4.0a	288 ± 4	267 ± 9	80.0 ± 13.9a	0.035 ± 0.002a	0.560 ± 0.259a
	*T*_dyn_	2.18 ± 0.08a	3.63 ± 0.38b	28.7 ± 3.7ab	45.5 ± 4.9b	271 ± 13	241 ± 13	24.9 ± 14.4b	0.046 ± 0.005a	0.281 ± 0.026ab
	*T*_40_	1.40 ± 0.12b	3.50 ± 0.44b	21.0 ± 4.1b	42.7 ± 5.5b	268 ± 12	241 ± 11	91.9 ± 17.6a	0.017 ± 0.005b	0.195 ± 0.034b
*S. leprosula*	*T*_30_	2.20 ± 0.07a	8.51 ± 0.57a	58.6 ± 8.9a	146.0 ± 14.0a	323 ± 10a	279 ± 8a	130.5 ± 22.3a	0.065 ± 0.004a	0.270 ± 0.036
	*T*_dyn_	2.16 ± 0.17a	5.45 ± 0.56b	55.4 ± 8.6a	72.2 ± 16.8b	323 ± 12a	237 ± 14b	29.7 ± 5.3b	0.074 ± 0.008a	0.189 ± 0.037
	*T*_40_	1.27 ± 0.20b	5.35 ± 0.53b	23.2 ± 4.5b	65.1 ± 11.3b	288 ± 6b	234 ± 13b	233.1 ± 15.7c	0.023 ± 0.004b	0.275 ± 0.135
*N. heimii*	*T*_30_	1.70 ± 0.14a	3.90 ± 0.45a	23.7 ± 3.0	49.9 ± 5.0a	274 ± 10	257 ± 5a	203.3 ± 65.2ab^†^	0.032 ± 0.007a	0.158 ± 0.053
	*T*_dyn_	1.96 ± 0.10a	2.91 ± 0.30ab	23.5 ± 4.6	31.0 ± 2.7b	257 ± 18	226 ± 7b	33.8 ± 27.2a^†^	0.060 ± 0.007b	0.516 ± 0.401
	*T*_40_	1.18 ± 0.13b	2.74 ± 0.27b	16.7 ± 2.0	28.0 ± 3.0b	264 ± 16	219 ± 5b	174.1 ± 20.3b^†^	0.022 ± 0.009a	0.078 ± 0.024
*L. senegalensis*	*T*_30_	1.90 ± 0.06a	4.83 ± 0.43	34.7 ± 7.9	63.9 ± 8.8	284 ± 17	255 ± 9	106.3 ± 20.3a^†^	0.046 ± 0.005a	0.120 ± 0.029
	*T*_dyn_	2.07 ± 0.08a	3.91 ± 0.48	38.2 ± 9.3	45.8 ± 9.1	290 ± 16	227 ± 11	15.0 ± 2.7b^†^	0.069 ± 0.008b	0.096 ± 0.021
	*T*_40_	1.42 ± 0.10b	3.78 ± 0.40	20.5 ± 3.7	46.6 ± 8.9	264 ± 10	235 ± 11	137.7 ± 3.0a^†^	0.022 ± 0.004c	0.114 ± 0.017

Shown are data recorded during gas exchange only measurements. Values are the means of five to six individual seedlings for each species ( ± standard error). Different letters following means indicate significant difference across different temperature conditions within each species, according to a LSD test conducted at P = 0.05 level. Absence of letters denotes absence of significant difference.

^†^Statistical analysis using one-way ANOVA and Dunnett’s T3 test.

Photosynthetic rate increased faster under *T*_dyn_ than either *T*_30_ or *T*_40_ and showed an overshoot within 10 min after light intensity increased. *A*_f_ under *T*_dyn_ was similar to that under *T*_40_. The time required to reach 50% of full photosynthetic induction (IT_f50%_) under *T*_dyn_ was 69–86% lower and 73–89% lower than that under *T*_30_ and *T*_40_, respectively (**Table 1**). The difference in ${\frac{dA}{dt}}_{\max}$ between *T*_30_ and *T*_dyn_ was significant in the shade-tolerant species.

Stomatal conductance before and at the end of induction decreased in all species under *T*_40_ compared to those under *T*_30_ (**Table 1**). The maximum rate of increase in *g*_sw_ was larger under *T*_30_ than *T*_dyn_, except for *N. heimii*. A larger depletion in *C*_i_ during induction was observed under *T*_dyn_ than *T*_30_ and *T*_40_ in all species (**Figure 1**).

### Photosynthetic Sub-Processes Under Different Temperature Conditions

**Figure 2:**
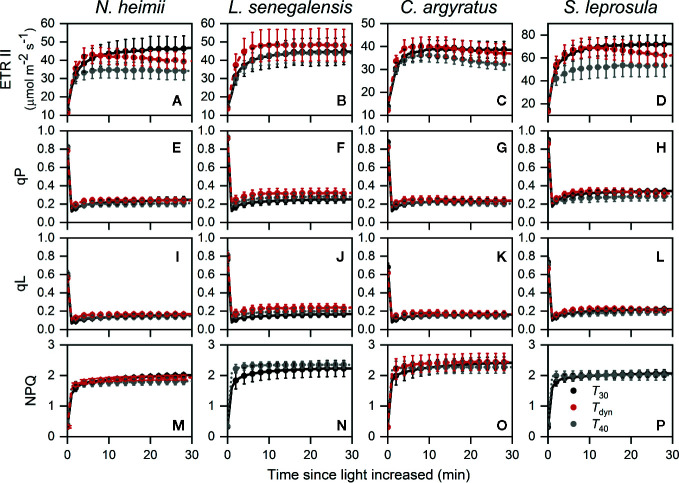
Time courses of ETR II **(A–D)**, qP **(E–H)**, qL **(I–L)**, and NPQ **(M–P)** during photosynthetic induction in *N. heimii* **(A, E, I, M)**, *L. senegalensis* **(B, F, J, N)**, *C. argyratus* **(C, G, K, O)**, and *S. leprosula* **(D, H, L, P)**. Values are the means ( ± SE) of three to six individual seedlings for each species under constant 30°C (*T*_30_) and 40°C (*T*_40_) and dynamic temperature condition (*T*_dyn_). NPQ in *L. senegalensis* and *S. leprosula* was not shown due to insufficient replicates (n < 3, see *Materials and Methods*). ETR II, electron transport rate of photosystem II; qP and qL are photochemical quenching based on the puddle and the lake model, respectively; NPQ, non-photochemical quenching.

The time required for ETR II to reach 50% of full induction (ETR_50%_) was 17–44% lower under *T*_dyn_ than *T*_30_ (**Table 2**). ETR II reached a maximum within 10 min and decreased afterward under *T*_dyn_ (**Figure 2**). The dynamics of qP and qL were similar among the three temperature conditions. In comparison with *T*_30_, NPQ increased faster under *T*_40_ in all species and under *T*_dyn_ in *N. heimii* and *C. argyratus*.

**Table 2:** Parameters of the time courses of ETR II and *V*_c_ during photosynthetic induction since the increase in irradiance from 50 to 1000 μmol m^-2^ s^-1^ in four tropical woody species under constant 30°C (*T*_30_), 40°C (*T*_40_), and dynamic temperature condition (*T*_dyn_).

Species abbreviation	Temperature condition	ETR_f_ (μmol m^-2^ s^-1^)	ETR_m_ (μmol m^-2^ s^-1^)	*V*_c_,_f_ (μmol m^-2^ s^-1^)	ETR_50%_(s)	τ_Rubisco_(s)
*C. argyratus*	*T*_30_	38.5 ± 3.1	39.8 ± 3.3	34.3 ± 3.0a	78.2 ± 9.4	73.2 ± 7.2
	*T*_dyn_	36.9 ± 3.6	40.7 ± 3.8	45.0 ± 3.3b	64.7 ± 2.4	87.8 ± 9.7
	*T*_40_	32.1 ± 2.3	36.6 ± 2.7	43.5 ± 2.1b	75.5 ± 10.5	114.4 ± 21.1
*S. leprosula*	*T*_30_	72.4 ± 7.1	72.7 ± 7.1	51.8 ± 4.4	92.6 ± 16.9 ^†^	139.6 ± 20.6ab
	*T*_dyn_	61.9 ± 8.4	69.7 ± 7.9	57.2 ± 9.0	72.9 ± 5.2 ^†^	117.9 ± 13.5a
	*T*_40_	53.1 ± 8.9	54.5 ± 8.7	57.2 ± 9.3	78.8 ± 32.4 ^†^	253.1 ± 68.2b
*N. heimii*	*T*_30_	46.6 ± 6.5	47.4 ± 6.4	37.2 ± 4.5	107.5 ± 17.9a	248.9 ± 48.9
	*T*_dyn_	39.4 ± 3.9	43.5 ± 4.0	44.2 ± 5.5	60.1 ± 4.1b	170.6 ± 10.3
	*T*_40_	34.2 ± 4.4	35.9 ± 4.5	37.6 ± 4.8	43.3 ± 9.0b	232.1 ± 30.4
*L. senegalensis*	*T*_30_	48.6 ± 6.9	49.6 ± 7.0	45.8 ± 5.2	120.9 ± 11.0a	150.2 ± 25.7ab ^†^
	*T*_dyn_	48.4 ± 7.5	49.0 ± 7.5	63.7 ± 8.4	80.6 ± 2.0b	111.6 ± 3.2a ^†^
	*T*_40_	47.0 ± 4.3	47.7 ± 4.5	61.3 ± 4.5	123.7 ± 8.9a	214.0 ± 18.3b ^†^

ETR_f_ and V_c,f_, ETR II and V_c_ reached at the end of photosynthetic induction, respectively; ETR_m_, maximum ETR II reached during photosynthetic induction; ETR_50%_, the time required for ETR II to reach 50% of full induction; τ_Rubisco_, the apparent time constant of Rubisco activation. Estimation was based on data recorded during chlorophyll flourescence measurements. Values are means of four to six individual seedlings for each species ( ± standard error). Different letters following means indicate significant difference across different temperature conditions within each species, according to a LSD test conducted at P = 0.05 level. Absence of letters denotes absence of significant difference.

^†^Statistical analysis using one-way ANOVA and Dunnett’s T3 test.

Steady-state *V*_c_ reached at the end of induction was higher under *T*_dyn_ and *T*_40_ than that under *T*_30_ (**Table 2**). The time constants of Rubisco activation were larger under *T*_40_ in all species, except for a small decrease in *N. heimii*. In comparison with *T*_30_, τ_Rubisco_ decreased under *T*_dyn_ in all species, except for a small increase in *C. argyratus*.

### Primary Limiting Factor During Photosynthetic Induction

**Figure 3:**
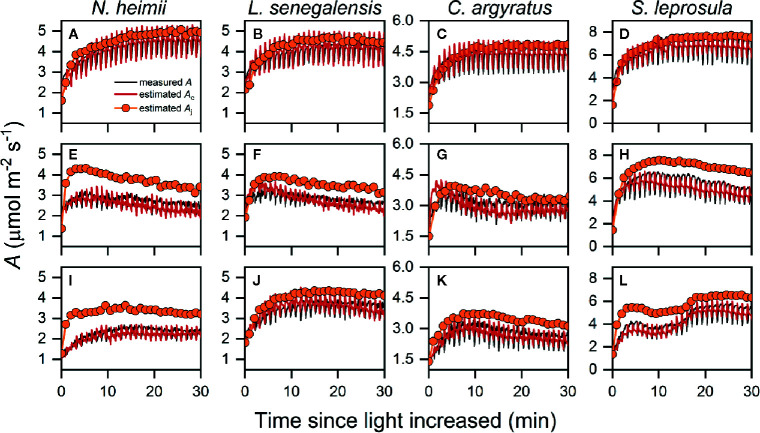
Representative time courses of measured *A* (open symbols), estimated *A*_c_ (red solid line), and estimated *A*_j_ (orange symbols with solid line) during photosynthetic induction in *N. heimii* **(A, E, I)**, *L. senegalensis* **(B, F, J)**, *C. argyratus* **(C, G, K)**, and *S. leprosula* **(D, H, L)** under constant 30°C [*T*_30_ **(A–D)**] simulated dynamic temperature [*T*_dyn_ **(E–H)**] and constant 40°C condition [*T*_40_ **(I–L)**], respectively. Measured *A* were those simultaneously recorded during chlorophyll fluorescence measurements. Estimated *A*_c_ and *A*_j_ were the potential *A* supported by *V*_c_ and ETR II, respectively. Periodic oscillations of *A* were inevitable due to the periodic dark pulses necessary for determining fluorescence yield.

**Figure 4:**
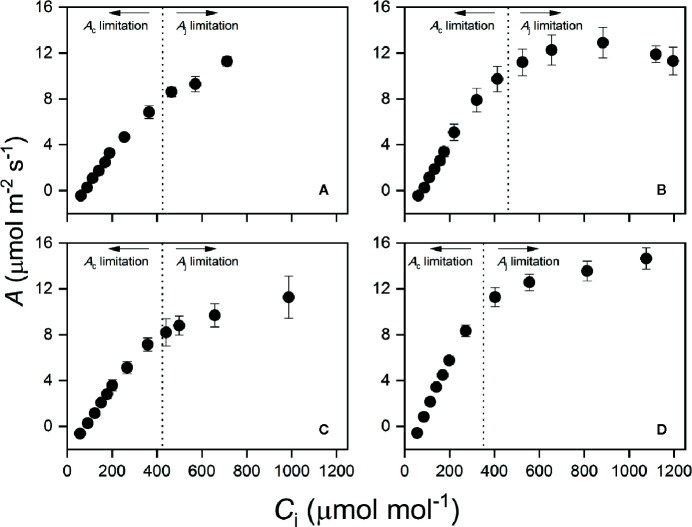
Photosynthetic CO_2_ response curve at high temperature (*T*_40_) in *N. heimii* **(A)**, *L. senegalensis* **(B)**, *C. argyratus* **(C)**, and *S. leprosula* **(D)**. Assimilation rate (*A*) was recorded under 1000 μmol m^-2^ s^-1^ at an average leaf temperature of 36.7°C.The *x*-intercept of vertical dotted lines represents the averaged transition CO_2_ concentration in each species, above which the primary limitation imposed on photosynthesis switched from *A*_c_ to *A*_j_. Values are the means ( ± SE) of five to six individual seedlings for each species. *A*, assimilation rate; *C*_i_, intercellular CO_2_ concentration.

As shown in **Figure 3**, estimated *A*_c_ matched the time course of measured *A*. We noted that *A* was limited by *A*_j_ only for the first several min (**Figure S3**), after which *A* was limited by *A*_c_ instead. The averaged time length of *A*_j_ limitation ranged from 1.4 to 2.7 min under *T*_30_, while the rest of photosynthetic induction was occupied by *A*_c_ limitation. Limitation from *A*_c_ almost dominated the entire induction process under *T*_40_ (**Figure S3**). This was consistent with CO_2_ response curves obtained at *T*_40_, as the transition point between Rubisco and RuBP regeneration limitation was much higher than transient *C*_i_ during induction in all species (**Figure 4**).

### Carbon Gain

**Figure 5:**
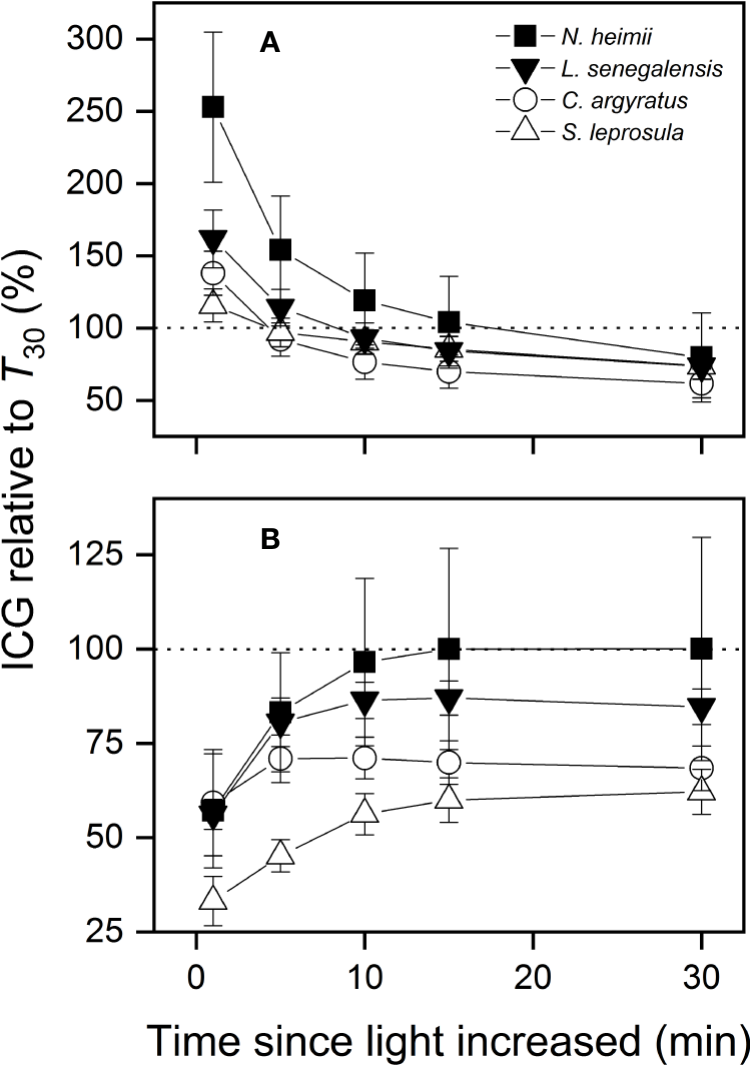
ICG under simulated dynamic temperature condition [*T*_dyn_ **(A)**] and under constant 40°C [*T*_40_ **(B)**] relative to that under constant 30°C (*T*_30_) as a function of the time since light increased in tree seedlings of four tropical woody species. Shown are data recorded during gas exchange only measurements. The dotted lines indicate equal amount of ICG between two temperature conditions. Open and closed symbols represent data from shade-intolerant and shade-tolerant species, respectively. Values are the means ( ± SE) of five to six individual seedlings for each species. No significant differences were found across species at *P* = 0.05 level.

ICG within the first 5 min under *T*_40_ was 45–83% of that under *T*_30_ (**Figure 5**). However, ICG within the first minute increased by 38–153% under *T*_dyn_ compared to that under *T*_30_. The differences in ICG between *T*_dyn_ and *T*_30_ decreased as the integration interval increased. ICG over 30 min (ICG_30min_) was 20–38% lower under *T*_dyn_ than that under *T*_30_. The shade-tolerant species showed larger increments in ICG under *T*_dyn_ and smaller decreases under both *T*_dyn_ and *T*_40_ than the shade-intolerant species.

### The Effects of Species-Specific Shade Tolerance and Growth Light Environment

In comparison with *T*_30_, increments in ${\frac{dA}{dt}}_{\max}$ and ICG_1min_ under *T*_dyn_ were significantly related to species-specific shade tolerance (**Table 3**). The decrease in IT_f50%_ was related to individual averaged DLI, as seedlings with low DLI showed greater reduction in ETR_f_ than those with high DLI (**Figure S4**).

**Table 3:** The influences of species-specific shade tolerance (*S*) and average DLI on the differences in induction responses between *T*_30_ and *T*_dyn_.

	Factors		
	Species-specific shade tolerance (*S*)	Average daily light integral (DLI)	*S* × DLI
IT_f50%_	1.399	1.600	2.664
${\frac{dA}{dt}}_{\max}$	5.731*	0.073	0.334
ETR_50%_	4.032	3.698	4.812*
*τ*_Rubisco_	0.054	0.012	0.533
ICG_1min_	5.455*	0.444	0.006
*A*_f_	1.799	0.484	1.311
*g*_sw,f_	1.217	0.804	1.044
ETR_f_	0.141	14.052**	0.329
ICG_30min_	0.922	0.041	0.241

The differences in induction responses were represented as the percentage change of a parameter. Shown are F statistics followed by significance symbols, which are *P < 0.05 and **P < 0.01 respectively.

## Discussion

### A Gradual Increase in Leaf Temperature Affects Photosynthetic Induction Process

Photosynthesis consists of a number of temperature-dependent biochemical processes (Berry and Björkman, 1980), and the induction process of photosynthesis thus depends on temperature. Recent studies showed that photosynthetic induction can be greatly altered by steady-state environmental temperature (Kaiser et al., 2017; Wachendorf and Küppers, 2017). It is however important to know how changing leaf temperature, accompanied with light changes, would affect photosynthetic induction rate. By comparing gradually increasing leaf temperature with two constant leaf temperatures after an increase in light, it is evident that an elevating leaf temperature from 30 to 40°C accelerates photosynthetic rate at the early-stage induction more than the two extreme constant temperatures of 30 and 40°C (**Figure 1**). This conclusion can be confirmed by the smaller IT_f50%_ and larger ${\frac{dA}{dt}}_{\max}$ (**Table 1**). The increase in simulated ETR at the early stage of the induction response also supports the conclusion (**Figure 2**). It should be also noticed that photosynthetic rate reached the steady-state much faster under the gradual increasing leaf temperature than either constant leaf temperatures, particularly in the shade-tolerant species (**Figure 1**). A full induction state of photosynthetic rate was achieved (within 2–3 min often) even before the leaf temperature reached its steady-state (about 10 min). This fact may indicate that a combined effect of changing leaf temperature, associated with an increase in light, on photosynthetic induction could include some different thermal processes rather than only under constant temperature conditions, which, to our knowledge, is being observed for the first time and deserves further clarification.

### Factors Involved in the Induction Process Under Different Temperature Conditions

During the first several min after an increase in light intensity, the increase in photosynthetic rate is often constrained by RuBP regeneration, which is further limited by ETR, light activation of Rubisco, and stomatal opening (Way and Pearcy, 2012; Kaiser et al., 2015; Yamori et al., 2020). All these factors are thermal sensitive, but the time constants of temperature and light stimulations could be considerably different (Leakey et al., 2003; Kaiser et al., 2017; Wachendorf and Küppers, 2017). It is difficult to elucidate individual effects of these factors only based on the gas change and chlorophyll fluorescence observations in this study. We tried to address how these factors contribute to photosynthetic induction under *T*_dyn_ using photosynthetic limitation analysis.

The acceleration of linear electron transport between photosystem II and I plays an evident role in the acceleration of early-stage induction of photosynthetic rate after increase of light, particularly in the shade-intolerant species (**Table 2**). In this study, the limitation of *A*_j_ dominates over the first 4–5 min under *T*_30_ (**Figure 3** and **Figure S3**), which was longer than those reported for soybean before (Sassenrath-Cole and Pearcy, 1992; Way and Pearcy, 2012). Crop plants grown under controlled environments may have higher RuBP concentration and/or higher activation rate of RuBP regeneration in comparison with plants growing within tropical forests. Decreased ETR_50%_ under *T*_dyn_ also indicated that accelerated induction of ETR was related to faster photosynthetic induction at the early stage under *T*_dyn_. Constant temperatures strongly affect RuBP regeneration during photosynthetic induction process (Kaiser et al., 2017). Thus, accelerated induction of ETR II is expected to benefit faster relaxation of limitation through RuBP regeneration process.

An increase in leaf temperature will result in increases in VPD in natural environment. Changes in VPD will affect photosynthetic induction by itself. For example, an increase in VPD reduced *g*_sw_ and thus increased diffusional limitation (Kaiser et al., 2017). On the other hand, when VPD was held constant, *g*_sw_ and *C*_i_ would increase with increasing *T*_leaf_ (Urban et al., 2017). In our study, if we assume that *g*_sw_ and *C*_i_ should remain the same as those reached under *T*_30_, then *A*_f_ under *T*_dyn_ would increase by 16% on average. If we focus on the early-stage of induction, then effects of changes in VPD can be neglected since stomatal opening and photosynthetic induction didn’t change much by VPD at this stage (Tinoco-Ojanguren and Pearcy, 1993; Kaiser et al., 2017). Therefore, concurrent increases in VPD with rising *T*_leaf_ will not significantly change our current conclusion in this study.

The overshoots during photosynthetic induction under *T*_dyn_ may be due to inhibition of some physiological processes by high VPD and *T*_leaf_. At the early-stage of induction when VPD and *T*_leaf_ were not so high, Rubisco was activated and stomata gradually opened. As VPD and *T*_leaf_ rose over a critical point, *g*_sw_ (**Figures 1E–H**), ETR II (**Figures 2A–D**), and possibly activation state of Rubisco (Yamori et al., 2006; Scafaro et al., 2016; Busch and Sage, 2017) decreased and thus *A* decreased. Nonetheless, the overshoots need to be clarified in the future.

### Photosynthetic Limitation Analysis

As discussed above, we determined the limiting process imposed on photosynthetic induction by comparing *A*_c_ and *A*_j_ after Farquhar et al. (1980). The classic photosynthetic limitation analysis defines photosynthetic limitation as a reduction in actual transient *A* compared with that estimated if biochemical or diffusional limitation was removed in one step. On the contrary, a stepwise method, which compares previous and subsequent photosynthesis state, produces smaller error than the one-step method, especially when time intervals between two states are small enough (Deans et al., 2019). The limitation analysis developed in this study is a stepwise method. Dynamic *A*-*C*_i_ analyses use high time-resolution dynamics of *V*_c_ and *J* by constructing induction curves at a wide range of different CO_2_ concentrations (Soleh et al., 2016; Taylor and Long, 2017; Salter et al., 2019). This method is time-consuming and risky due to the dependency of Rubisco activation state on CO_2_ concentration (Mott and Woodrow, 1993; Woodrow et al., 1996; Tomimatsu et al., 2019). Our method provides a compromise between convenience and accuracy and can be promoted with higher time-resolution fluorescence signals for both PSI and PSII.

Our observations showed that *T*_leaf_ changed by <0.2°C/s for the first min and <0.05°C/s for the rest of induction (**Figure 1**). Such changes in *T*_leaf_ should result in small changes in the steady-state *R*_L_ and Г^*^. Thus, assuming instantaneous response of both parameters imposed little influence (<0.1%) on estimated *A*_c_ or *A*_j_. The effect of a time lag in *K*_m_ response is also limited. If *K*_m_ changes by 50% of difference between two consecutive steady-states, *A*_c_ under *T*_dyn_ changes by less than 5%, in comparison with that assuming instantaneous response of *K*_m_. A decrease in leaf absorptance and/or fraction of absorbed light allocation to PSII is likely to occur when a shaded leaf is exposed to high light for long (Davis et al., 2011; Dutta et al., 2015; Mekala et al., 2015). A survey from 24 species indicates that leaf absorptance of PAR decreased by ~5% after 2 h exposure to high light (Davis et al., 2011), which alone may lead to an overestimation of *A*_j_ by ~5% and hence underestimation of *A*_j_ limitation. Simulation from Morales et al. (2018) also indicate small influences on *A* from changes in leaf absorptance. If allocation fraction should be 0.45 from the very beginning of induction, then *A*_j_ decreased by ~10%. This would increase the duration of *A*_j_ limitation, thus the dominant role of *A*_j_ over the early-stage of induction still holds.

### Ecological Consequences of Changing Leaf Temperature With PAR

Concurrent change of leaf temperature with PAR may play an important role in leaf carbon uptake and energy balance under temporally variable light environments. Leakey et al. (2003) reported a decrease in ICG in *S. leprosula* seedlings at elevated constant temperature. In this study, we demonstrate that leaf carbon gain is enhanced within the first several min under *T*_dyn_, although photosynthetic rate was depressed at the steady-state under 40°C (**Table 1**). Since most sunflecks occurring under dense forest canopies last only a few min (Pearcy, 1983; Chazdon and Pearcy, 1986b), the acceleration of photosynthetic rate accompanied with the increase in leaf temperature at the early stage of the induction suggests that short sunflecks may contribute more leaf carbon gain than previously estimated under constant temperature.

Moreover, it is still debated whether shade-tolerant species can use sunflecks more efficiently than shade-intolerant species (Naumburg and Ellsworth, 2000; Rijkers et al., 2000; Way and Pearcy, 2012). However, the argument is based on the knowledge obtained only under single constant temperature. When taking variation of leaf temperature into account, more leaf carbon gain may be achieved for shade-tolerant species because these species showed higher acceleration of photosynthetic rate than the shade-intolerant species under the changing leaf temperature in this study.

ecent studies suggest that shade-intolerant species from tropical regions have higher photosynthetic temperature optimum, lower *T*_leaf_, and a wider temperature range for photosynthesis (Cheesman and Winter, 2013; Slot and Winter, 2017a; Slot and Winter, 2017b) and thus seem more competitive than shade-tolerant species in a warming world. A less strong reduction in ICG found in the shade-tolerant species under *T*_dyn_ and *T*_40_ (**Figure 5**), however, provides some contrasting evidence. Detailed assessments on photosynthetic response and energy balance under dynamic environments, particularly under changing light and temperature conditions, are urgently needed to understand the effect of climate change on plants in tropical forests.

## Conclusion

We provide the first evidence that increase in leaf temperature, associated with increase in light, accelerates photosynthetic rate at the early stage of induction process. We further demonstrated that the acceleration is likely to be mainly due to accelerated induction of ETR II. These results extend our understanding of dynamic photosynthesis to cover the effects of concurrent changes in leaf temperature and light. However, there are a number of limitations in this preliminary study, and further studies are needed to understand physiological controls of the concurrent changes, particularly in relation to leaf energy budget.

## Data Availability Statement

The raw data supporting the conclusions of this article will be made available by the authors, without undue reservation.

## Author Contributions

H-XK and Y-HT contributed to conception and design of the study. H-XK performed the experiments and the statistical analysis. H-XK wrote the manuscript. X-GZ, WY, and Y-HT provided editorial and scientific advice. All authors contributed to the article and approved the submitted version.

## Funding

This study was funded by the Key Research of Plant Functional Ecology Program of Peking University (no. 7101302307). This work was supported in part by JSPS KAKENHI (grant numbers JP16H06552, JP18H02185 and JP18KK0170 to W.Y).

## Conflict of Interest

The authors declare that the research was conducted in the absence of any commercial or financial relationships that could be construed as a potential conflict of interest.
