# Supplementary material for: Concurrent Increases in Leaf Temperature With Light Accelerate Photosynthetic Induction in Tropical Tree Seedlings
Source: Front Plant Sci. 2020 Aug 7;11:1216. doi: 10.3389/fpls.2020.01216 (PMC7427472; doi:10.3389/fpls.2020.01216)
Supplement: Supplementary file 2 [file Table_2.docx]

**Supplementary File S2**

**Part 1. Calibrating ETR II estimated from chlorophyll fluorescence**

Although ETR II has been shown to correlate well with linear electron flow calculated from measured O_2_ evolution rate in some cases, it is not an accurate estimation, see (Kalaji et al., 2017) for more detail. This deviation in ETR II led to the difference between measured *A* and estimated *A*_j_. In some cases, this difference was as large as 30% of transient *A* (Figure S1). This should not be true, as the coordination theory of leaf photosynthesis states that plant actively regulated to achieve the balance between *A*_c_ and *A*_j_ in response to environmental conditions (Chen, Reynolds, Harley, & Tenhunen, 1993), such as *T*_30_ applied in this study. Therefore, we introduced a parameter to ensure that *A*_j_ matches *A*_c_ at the end of induction (for *T*_30_ only) and then applied it to all transient *A*_j_, including *T*_dyn_ and *T*_40_.

However, the coordination theory has been validated for growth conditions only (Maire et al., 2012) and may not apply for non-steady-state and potential stressful conditions, like *T*_dyn_ and *T*_40_ (Walker et al., 2014). A recent report using dynamic *A*-*C*_i_ method also suggests that transient *A*_j_ would be higher than transient *A*_c_ for almost entire period of induction at quasi growth condition (Taylor & Long, 2017). Therefore, differences between transient *A*_c_ and *A*_j_ under *T*_dyn_ and *T*_40_ are reasonable.

**Part 2. Modelling the midway decrease in assimilation rate during induction**

In some induction curves obtained under *T*_dyn_ and *T*_40_, *A* decreased within several minutes since the onset of the lightfleck. We first assumed no decrease in *V*_c_ occurred during induction and then calculated *A*_c_ as described in the article:

$A_{c}(t)=V_{c}(t)\frac{C_{i}(t)-\Gamma^{*}(T)}{C_{i}(t)+K_{m}(T)}-R_{L}(T)$ (S1)

$V_{c}\left( t \right)=V_{c,f}-\left( V_{c,f}-V_{c,ini} \right)*exp(-t/\tau_{\mathrm{Rubisco}})$ (S2)

The results were compared against measured *A*, as shown in Figure S2. It is clear that *A*_c_ were much larger than measured *A* during the late phase of induction. Such deviations went against the widely reported limitation by Rubisco-capacity on photosynthesis at high temperatures in literatures (Hikosaka, Ishikawa, Borjigidai, Muller, & Onoda, 2006; Sage & Kubien, 2007; Yamori, Hikosaka, & Way, 2014), confirmed by our analysis of the photosynthetic CO_2_ response curves (Fig. 4 in the text).

Thus, midway deactivation of Rubisco was deemed likely. We divided induction curves into two parts, an increasing part and a decreasing part. Each part was fitted after the method proposed by (Woodrow & Mott, 1989) to obtain $\tau_{\mathrm{Rubisco}}$. For the increasing parts, $V_{c,ini}$ and $V_{c,f}$ were estimated from data recorded prior to the increase in irradiance and at the end of the part. For the decreasing parts, $V_{c,ini}$ were set to equivalent to $V_{c,f}$ of the increasing part and $V_{c,f}$ were estimated from data at the end of induction.

**Figures**

**

**

**Figure S1.** Exemplar time courses of measured *A* and estimated *A*_j_ without calibration in a tree seedling of *S. leprosula*. Measured *A* were simultaneously recorded during chlorophyll fluorescence measurements. Estimated *A*_j_ was the potential *A* supported by transient ETR II. Periodic oscillations of *A* were inevitable, due to the periodic dark pulses necessary for determining fluorescence yield.

**
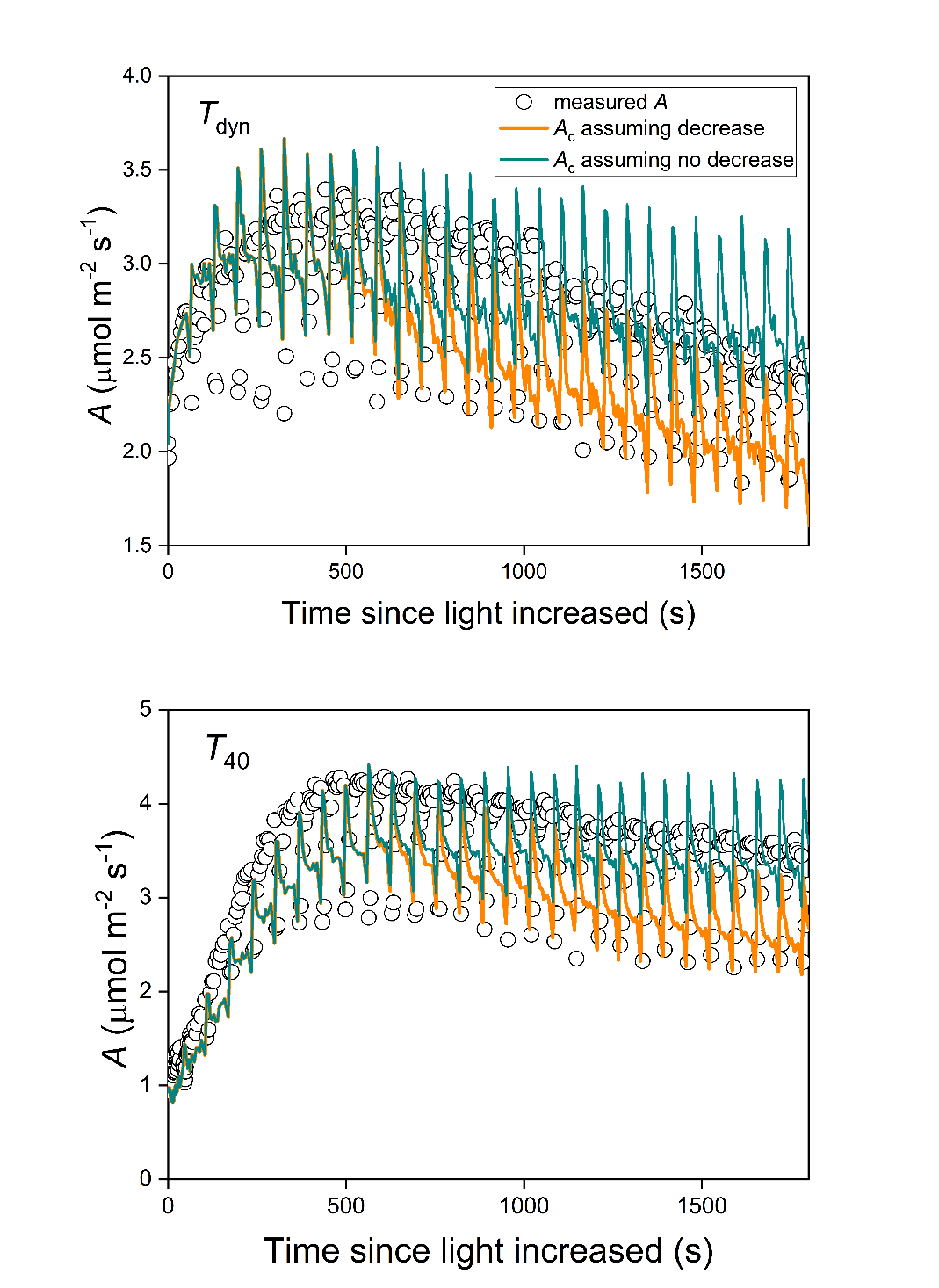
**

**Figure S2.** Exemplar time courses of measured *A* and estimated *A*_c_ with and without assuming decrease in *V*_c_ during photosynthetic induction under simulated dynamic temperature (*T*_dyn_, upper) and constant 40 °C condition (*T*_40_, lower), respectively. Measured *A* were simultaneously recorded during chlorophyll fluorescence measurements. Estimated *A*_c_ was the potential *A* supported by transient *V*_c_, respectively. Periodic oscillations of *A* were inevitable, due to the periodic dark pulses necessary for determining fluorescence yield.

**Reference**

Chen, J. L., Reynolds, J. F., Harley, P. C., & Tenhunen, J. D. (1993). Coordination theory of leaf nitrogen distribution in a canopy. *Oecologia, 93*, 63-69.

Hikosaka, K., Ishikawa, K., Borjigidai, A., Muller, O., & Onoda, Y. (2006). Temperature acclimation of photosynthesis: mechanisms involved in the changes in temperature dependence of photosynthetic rate. *J Exp Bot, 57*(2), 291-302. doi:10.1093/jxb/erj049

Kalaji, H. M., Schansker, G., Brestic, M., Bussotti, F., Calatayud, A., Ferroni, L., . . . Baba, W. (2017). Frequently asked questions about chlorophyll fluorescence, the sequel. *Photosynth Res, 132*(1), 13-66. doi:10.1007/s11120-016-0318-y

Maire, V., Martre, P., Kattge, J., Gastal, F., Esser, G., Fontaine, S., & Soussana, J. F. (2012). The coordination of leaf photosynthesis links C and N fluxes in C_3_ plant species. *PLoS One, 7*(6), e38345. doi:10.1371/journal.pone.0038345

Sage, R. F., & Kubien, D. S. (2007). The temperature response of C_3_ and C_4_ photosynthesis. *Plant, Cell and Environ, 30*(9), 1086-1106. doi:10.1111/j.1365-3040.2007.01682.x

Taylor, S. H., & Long, S. P. (2017). Slow induction of photosynthesis on shade to sun transitions in wheat may cost at least 21% of productivity. *Philosophical Transactions of the Royal Society B: Biological Sciences, 372*(1730). doi:10.1098/rstb.2016.0543

Walker, A. P., Beckerman, A. P., Gu, L., Kattge, J., Cernusak, L. A., Domingues, T. F., . . . Woodward, F. I. (2014). The relationship of leaf photosynthetic traits - *V*_cmax_ and *J*_max_ - to leaf nitrogen, leaf phosphorus, and specific leaf area: a meta-analysis and modeling study. *Ecol Evol, 4*(16), 3218-3235. doi:10.1002/ece3.1173

Woodrow, I. E., & Mott, K. A. (1989). Rate limitation of non-steady-state photosynthesis by ribulose-1,5-bisphosphate carboxylase in spinach. *Australian J Plant Physiol, 16*, 487-500.

Yamori, W., Hikosaka, K., & Way, D. A. (2014). Temperature response of photosynthesis in C_3_, C_4_, and CAM plants: temperature acclimation and temperature adaptation. *Photosynthe Res, 119*(1-2), 101-117. doi:10.1007/s11120-013-9874-6
