# Supplementary material for: Concurrent Increases in Leaf Temperature With Light Accelerate Photosynthetic Induction in Tropical Tree Seedlings
Source: Front Plant Sci. 2020 Aug 7;11:1216. doi: 10.3389/fpls.2020.01216 (PMC7427472; doi:10.3389/fpls.2020.01216)
Supplement: Supplementary file 3 [file Table_3.docx]

**Figure S1.** Representative diurnal trace of leaf temperature (*T*_leaf_) and photosynthetic active radiation after transmittance through the leaf (transmitted PAR) of a *Shorea leprosula* seedling in the forest floor of a lowland tropical rain forest, Pasoh, Malaysia. Leaf temperature was measured using a thermocouple touching the abaxial leaf surface. A PAR quantum sensor (MIJ-14PAR K2; Environmental Measurement Japan, Fukuoka, Japan) was levelled to horizontal and placed right under the leaf at a distance less than 1 cm. Data were recorded every second by a datalogger.
