# Supplementary material for: Concurrent Increases in Leaf Temperature With Light Accelerate Photosynthetic Induction in Tropical Tree Seedlings
Source: Front Plant Sci. 2020 Aug 7;11:1216. doi: 10.3389/fpls.2020.01216 (PMC7427472; doi:10.3389/fpls.2020.01216)
Supplement: Supplementary file 4 [file Table_4.docx]

**

**

**Figure S2.** Representative diurnal traces of modelled leaf-to-air vapor pressure deficit (VPD_leaf-to-air_). Leaf temperature was assumed to be consistently equal to measured air temperature (*T*_air_, light grey dotted line) and higher than *T*_air_ by 2°C (grey dashed line) or 5°C (black solid line). Leaf vapor pressure was assumed to be saturated and calculated after the equation adopted in LI-6800: $SVP=0.6135e^{\frac{17.502T}{240.97+T}}$, where SVP (kPa) is saturated vapor pressure at temperature *T* (°C). VPD_leaf-to-air_ was calculated as the difference between leaf vapor pressure and vapor pressure of the air, the latter of which was converted from *T*_air_ and measured relative humidity of the air.
