# Supplementary material for: Concurrent Increases in Leaf Temperature With Light Accelerate Photosynthetic Induction in Tropical Tree Seedlings
Source: Front Plant Sci. 2020 Aug 7;11:1216. doi: 10.3389/fpls.2020.01216 (PMC7427472; doi:10.3389/fpls.2020.01216)
Supplement: Supplementary file 5 [file Table_5.docx]

**

**

**Figure S3.** Time length of *A*_j_ limitation as the primary limiting factor on transient *A* during photosynthetic inductio under three different temeprature conditions among four tropical woody species. Values are mean of 3-5 individual seedlings for each species (± standard error). Different letters following means indicate significant (*P* < 0.05) difference across different temperature conditions within each species.
