# Supplementary material for: Concurrent Increases in Leaf Temperature With Light Accelerate Photosynthetic Induction in Tropical Tree Seedlings
Source: Front Plant Sci. 2020 Aug 7;11:1216. doi: 10.3389/fpls.2020.01216 (PMC7427472; doi:10.3389/fpls.2020.01216)
Supplement: Supplementary file 6 [file Table_6.docx]

**Figure S5.** The ratio of final steady state ETR II (ETR_f_) between *T*_dyn_ and *T*_30_ as a function of average daily light integral of each seedling. Open and closed symbols indicate data from the shade-intolerant and shade-tolerant seedlings, respectively. The solid line represents the linear regression for all seedlings.
